# The End of the Line: Can Ferredoxin and Ferredoxin NADP(H) Oxidoreductase Determine the Fate of Photosynthetic Electrons?

**DOI:** 10.2174/1389203715666140327113733

**Published:** 2014-06

**Authors:** Tatjana Goss, Guy Hanke

**Affiliations:** Department of Plant Physiology, Faculty of Biology and Chemistry, University of Osnabrück,11 Barbara Strasse, Osnabrueck, DE-49076, Germany

**Keywords:** Bundle sheath, cyclic electron flow, ferredoxin, FNR, mesophyll.

## Abstract

At the end of the linear photosynthetic electron transfer (PET) chain, the small soluble protein ferredoxin (Fd) transfers electrons to Fd:NADP(H) oxidoreductase (FNR), which can then reduce NADP^+^ to support C assimilation. In addition to this linear electron flow (LEF), Fd is also thought to mediate electron flow back to the membrane complexes by different cyclic electron flow (CEF) pathways: either antimycin A sensitive, NAD(P)H complex dependent, or through FNR located at the cytochrome b_6_*f* complex. Both *Fd* and *FNR* are present in higher plant genomes as multiple gene copies, and it is now known that specific Fd iso-proteins can promote CEF. In addition, FNR iso-proteins vary in their ability to dynamically interact with thylakoid membrane complexes, and it has been suggested that this may also play a role in CEF. We will highlight work on the different Fd-isoproteins and FNR-membrane association found in the bundle sheath (BSC) and mesophyll (MC) cell chloroplasts of the C4 plant maize. These two cell types perform predominantly CEF and LEF, and the properties and activities of Fd and FNR in the BSC and MC are therefore specialized for CEF and LEF respectively. A diversity of Fd isoproteins and dynamic FNR location has also been recorded in C3 plants, algae and cyanobacteria. This indicates that the principles learned from the extreme electron transport situations in the BSC and MC of maize might be usefully applied to understanding the dynamic transition between these states in other systems.

## THE END OF THE LINE – POSSIBLE FATES OF PHOTOSYNTHETIC ELECTRONS

In photosynthetic electron transfer (PET), electrons are originally generated by the splitting of water at photosystem II (PSII), transferred to the cytochrome b_6_*f* complex (Cytb_6_*f*) by plastoquinone (PQ), and from there through the thylakoid lumen to photosystem I (PSI), via plastocyanin (PC). The final events in PET involve the transfer of excited electrons from PSI to the small, soluble [2Fe-2S] protein ferredoxin (Fd) [1]. Fd was first identified in studies on nitrogen fixation in *Clostridium pasteuranium* [2] as a soluble, iron containing electron transfer factor, but in work on plant proteins this term has come to be almost exclusively associated with the ~10 kDa [2Fe-2S] proteins involved in photosynthesis. In linear electron flow (LEF) Fd is capable of transferring photosynthetically derived electrons to diverse Fd-dependent enzymes [3], and shows variable interaction with these different proteins [4, 5]. In this way, the physical properties of Fd help determine the flux of reducing power into different metabolic pathways [6]. 

The carbon fixing Calvin Benson cycle contains one reaction that requires photosynthetically generated electrons, the NADPH-dependent reduction of 1,3-diphospholycerate to glyceraldehyde 3-P, catalyzed by glyceraldehyde-3-P dehydrogenase. Electrons destined for carbon fixation must therefore be used to reduce NADP^+^ to NADPH, a reaction catalyzed by the enzyme Fd:NADP(H) oxidoreductase (FNR) [7]. The relatively high affinity of FNR for reduced Fd [8] therefore reflects its status as the premier conduit of electrons from reduced Fd into soluble metabolism, and the high priority placed on carbon fixation. In addition to facilitating the flux of electrons from the thylakoid membrane into soluble metabolism, Fd is an integral part of their return to the PQ pool in cyclic electron flow (CEF) [9]. CEF allows the generation of ΔpH without the net release of electrons from the membrane, and is essential for balancing the proportion of ATP:NADPH produced by PET, and therefore for normal plant growth [10]. Alternative mechanisms by which photosynthetic organisms can modulate the balance between NADPH and ATP production, in order to respond to the demands on their metabolism caused by changing environmental conditions and environment are well reviewed elsewhere [11], and include the malate valve [12] and the water-water cycle [13].

In the water-water cycle, an absence of oxidized Fd at PSI results in electron donation to O_2_, resulting in generation of superoxide (O_2_^∙-^) [14]. Because of the damaging nature of O_2_^∙-^, it is rapidly converted to H_2_O_2_ by superoxide dismutase enzymes. Peroxidase enzymes then reductively convert H_2_O_2_ to H_2_O and O_2_, using ascorbate as the electron donor [15, 16]. Ascorbate is regenerated using photosynthetic electrons, either directly via Fd, or by NADPH via reduced glutathione [17]. The water-water cycle therefore combines the consumption of electrons with electron flow through PET, and therefore proton pumping and ATP synthesis. 

In the absence of other acceptors, excited chlorophyll at the light harvesting complex of PSII can generate the extremely dangerous singlet oxygen radical [18]. Higher plants and algae prevent this through a variety of mechanisms referred to as non-photochemical quenching (NPQ) [19]. In this case, activity of PSII is down regulated through a combination of photoinhibition at PSII [20], state transitions [21] and photon quenching in the xanthophyll cycle [22]. During these circumstances, CEF takes on even greater importance. For example in high light, or under conditions of drought [23], when stomata are closed and there is therefore a lack of electron acceptors in CO_2_-assimilation, PSII activity is decreased by NPQ, while PSI is still relatively active in CEF. 

CEF pathways in photosynthetic organisms are well reviewed elsewhere [11, 24], and we will focus here only on the well characterized pathways of CEF involving Fd. The antimycin A sensitive pathway is regulated by the PGR5 protein [25], and involves transfer of electrons from Fd to the PQ pool by the protein PGRL1 [26] in a calcium dependent manner [27]. The PGR5 and PGRL1 proteins are absent from cyanobacterial genomes, and this pathway appears to have evolved in the algae [28] and is present in higher plants [25]. The NAD(P)H dehydrogenase (NDH) complex dependent pathway involves a homologue of the respiratory complex I, comprising multiple subunits [29]. Although genes for essential subunits appear to have been lost in some algae [30] and gymnosperms [31] the complex is an important part of angiosperm PET under high light conditions [32] and has also recently been shown to require Fd as a direct electron donor [33]. There is evidence that FNR may also be involved in CEF, based on biochemical and genetic data [34-36]. It has been suggested that FNR could either directly reduce the PQ pool using electrons from Fd [37], or act as a Fd binding site on the thylakoid membrane during CEF, as it has been found to associate with both the NDH complex [38], and the Cytb_6_*f* [39]. Cyanobacterial FNR contains a phycobilisome linker protein for attachement to the thylakoid [40], and was not found associated with the Cytb_6_*f* [39]*,* so such a pathway must have evolved after the loss of phycobilisomes during chloroplast evolution. Indeed, a complex of FNR, Cytb_6_*f,* PGRL1 and PSI was isolated from the alga *Chlamydomonas rheinhardtii*, and found to catalyze CEF [28], but this finding has not been replicated in higher plants [26]. It is possible that direct association of FNR with the Cytb_6_*f* , as identified in spinach [39] and maize [41] might catalyze Fd-dependent reduction of PQ via the Cytb_6_*f *in higher plants [35]. 

**Fig. (1):**
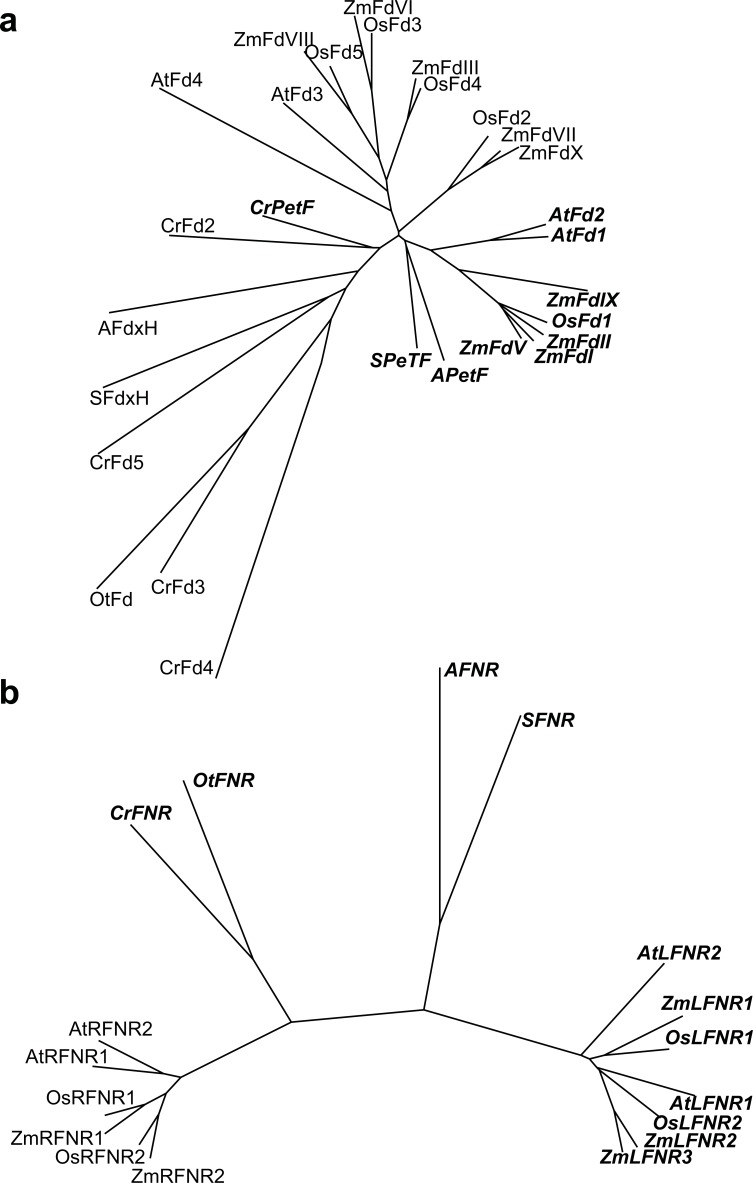
**Multiple gene copies encode the proteins at the end of the photosynthetic electron transport chain.** Phylogenetic tree showing diversity of **(a)**, Fd sequences and **(b)**, FNR sequences in photosynthetic organisms. Mature sequences aligned in Clustal Omega (http://www.ebi.ac.uk/Tools/msa/clustalo/) and phylogenetic trees drawn in Phylodendron (http://iubio.bio.indiana.edu/treeapp/treeprint-form.html). Sequences of transit peptides were excluded based on mature N-terminal sequence information. Proteins with known or inferred involvement in photosynthetic electron transport are in bold italics. Fd sequence assignments are as follows (*Synechocystis sp.* PCC 6803) SPetF, NP_442127.1; SFd2, NP_440517.1; SFdxH, NP_441872.1; (*Nostoc sp. *PCC 7120) APetF, NP_488188.1; AFdxH, NP_485473.1; (*Chlamydomonas reinhardtii*) CrPetF, XP_001692808.1; CrFd2, XP_001697912; CrFd3, XP_001691381.1; CrFd4, XP_001690910.1; CrFd5, XP_001691603; (*Oryza sativa*) OsFdI, NP_001060779.1; OsFdII, NP_001052738.1; OsFdIII, NP_001051821.1; OsFdIV, NP_001055675.1; OsFdV, NP_001172661.1; (*Arabidopsis thaliana*) AtFd1, NP_172565.1; AtFd2, NP_176291.1; AtFd3, NP_180320; AtFd4, NP_196562.1; (*Zea mays*) ZmFdI, P27787.1; ZmFdII, O80429; ZmFdIII, NP_001105346; ZmFdIV, NP_001158976; ZmFdV, ACA34366.1; ZmFdVI,ACG32559; ZmFdVII, NP_001130605; ZmFdVIII, NP_001150016.1; ZmFdIX, NP_001168703.1. Higher plant leaf and root FNR sequences are referred to in the figure as LFNR and RFNR for clarity, and FNR sequence assignments are as follows (*Zea mays*): ZmLFNR1, BAA88236; ZmLFNR2, BAA88237; ZmLFNR3, ACF85815; ZmRFNR1, ACG39703.1; ZmRFNR2, ACG35047.1 ; (*Oryza sativa*) OsLFNR2, Os02G0103800; OsLFNR1, OS06G0107700; OsRFNR1, OS03G0784700; OsFNR2, Os07g0147900; (*Arabidopsis thaliana*) AtLFNR1, AT5G66190; AtLFNR2, AT1G20020; AtRFNR2, AT1G30510; AtRFNR1, AT4G05390; (*Chlamydomonas reinhardtii*) CrFNR, XP_001697352.1; (*Ostreococcus taurii*) OtFNR, XP_003084170.1; (*Nostoc sp. *PCC 7120) AFNR, NP_488161.1; (*Synechocystis sp. *PCC 6803) SFNR, NP_441779.1.

The actions of Fd and FNR during the final steps of LEF therefore determine the immediate fate of photosynthetically generated electrons: will they be released into the stroma as NADPH [7] or direct Fd donation to other enzymes? [3], or will they be returned to the PQ pool in CEF? [9, 33] *Fd* is encoded as multiple copies in the genomes of most photosynthetic organisms (Fig. **1a**) [3], and even cyanobacteria contain additional copies of *Fd* genes as well as the primary photosynthetic *PetF.* For example, as can be seen in (Fig. **1a**), in the model organism *Synechocystis* sp. PCC6803 there are cDNA sequences encoding proteins for PetF (NP_442127.1), the heterotropic FdxH (NP_441 872.1) and the uncharacterized Fd2 (NP_440517.1). FNR is also present as multiple iso-proteins, but only in higher plants (Fig. **1B**). However, it has been demonstrated that the enzyme can be present in distinct locations, as both membrane bound and soluble pools in cyanobacteria [42] and higher plants [41, 43, 44]. Higher plants often contain multiple gene copies for both photosynthetic FNR (often termed leaf FNR, or LFNR) and non-photosynthetic FNR (often termed root FNR, or RFNR) [41, 44-46]. In heterotrophic plant tissues, essential Fd-dependent metabolism is supported by this root FNR in a reversal of the photosynthetic reaction, and also involves specific root type Fd [47]. These iso-proteins will not be discussed in this review, which focuses on photosynthetic electron channeling. We will specifically focus on how different Fd and FNR forms could be differentially involved in partitioning electrons into either LEF or CEF. This would therefore allow the plant to control electron partitioning into LEF and CEF by regulating the abundance of specific Fd and FNR iso-proteins. In this article we will discuss the evidence supporting this view, and highlight the remaining gaps in our understanding. 

## THE DIFFERENT FATE OF ELECTRONS IN MAIZE MESOPHYLL AND BUNDLE SHEATH CHLOROPLASTS

**Fig. (2):**
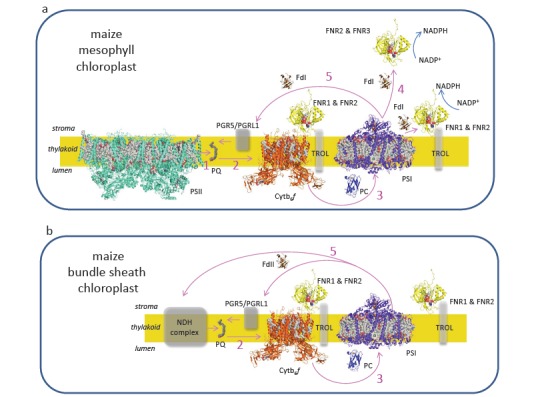
**Contributions of Fd and FNR to photosynthetic electron transport in (a) the mesophyll cell chloroplasts and (b) the bundle sheath cell chloroplasts of maize.** Protein structures shown where known, or grey boxes where not. Electron transfer reactions shown as labeled pink arrows. In the linear photosynthetic electron transport chain, water is split at photosystem II (PSII), releasing electrons that are accepted by plastoquinone (PQ) (1), which transfers them to the cytochrome b_6_*f* complex (Cytb_6_*f*) (2). Plastocyanin (PC) carries these electrons through the thylakoid lumen to photosystem I (PSI) (3), where they are donated to ferredoxin (Fd). Fd can donate these electrons to multiple enzymes, including Fd:NADP(H) oxidoreductase (FNR) (4), which then photoreduces NADP^+^. In addition to this linear electron flow (LEF), Fd may return electrons to the membrane via either PGRL1 (the antimycin A sensitive pathway) or the NAD(P)H complex (NDH) dependent pathway (5). Both linear and cyclic electron flow generate the pH gradient necessary for ATP synthesis, but only the linear path results in release of electrons into stromal metabolism. Maize bundle sheath cells have very high rates of cyclic electron flow. This is facilitated by the presence of very little active PSII, a Fd iso-protein (FdII) specific for the cyclic pathway, and elevated amounts of the NDH complex. Moreover, only two FNR iso-proteins, FNR1 and FNR2, are present, and these are tightly bound to the membrane by the thylakoid rhodanase like protein (TROL) and also associated with Cytb_6_*f*, although their precise role in cyclic electron flow remains to be established. By contrast, the mesophyll cells have abundant, active PSII and relatively low amounts of the NDH complex. In combination with the specific Fd iso-protein, Fd1, this facilitates LEF. In the mesophyll three FNR proteins are present, FNR1, FNR2 and FNR3. All of FNR3 and a large proportion of FNR2 is soluble, and presumably involved in linear NADP^+^ photoreduction.

In C4 photosynthesis, different steps in carbon fixation are divided between two different cell types, known as the mesophyll (MC) and bundle sheath (BSC) [48, 49]. Initially carbon is fixed into a C4 acid in the mesophyll cytosol (rather than a C3 sugar in the chloroplast, as occurs in standard, C3 photosynthesis), and then transported into the BSC for decarboxylation to yield a high CO_2_ environment around RuBisCO. C4 metabolism has arisen many different times in evolution, and plants vary in the form of carbon transported and the specific enzymes involved in decarboxylation [50]. The more efficient C fixation in C4 plants comes at the cost of two more ATP molecules per C fixed [49], and in order to compensate for the increased ATP/NADPH demand, it has been reported that these plants perform increased CEF [51, 52]. Plants in the *Flaveria* genus contain species that perform either C3, C4 or intermediate C3-C4 photosynthesis [53], and are therefore often used as a comparative model. Maize (*Zea mays*) is also a commonly used photosynthetic and biochemical model for C4 photosynthesis, and in both the C4-*Flaveria* and maize, decarboxylation of malate by NADP-malic enzyme in the BSC yields NADPH, decreasing the demand for LEF to photo-reduce NADP^+^. As well as the increased overall demand for ATP, the ratio for ATP/ NADPH requirement in the maize and *Flaveria* BSC is therefore particularly high. For this reason, chloroplasts in maize MC and BSC make an excellent comparative model of cells performing predominantly LEF and CEF respectively. The predominant PET pathways in maize MC and BSC are depicted in (Fig. **2**).

Indeed, it has been shown that both NDH-H (NDH-pathway) and PGR5 (antimycin A sensitive pathway) have enhanced abundance in the BSC of C4 performing *Flaveria *species [54]. Another study has shown that while the abundance of NDH complex subunits is specifically up-regulated in the BSC, PGR5 and PGRL1 are actually up-regulated in both cell types during the transition from C3 to C4 in *Flaveria* [55]. Although it is reported that the NDH complex, but not PGR5, has increased abundance in the BSC of maize [56], PGR5 is increased in abundance relative to the Cytb_6_*f* [54]. A comparison between maize BSC and MC chloroplasts therefore provides an excellent tool understanding whether Fd and FNR iso-protein diversity might contribute to the plants ability to control electron partitioning. This understanding can now be applied in attempting to understand the dynamic transition between LEF and CEF in C3 species, algae and cyanobacteria, where there are no separate cell types to aid identification of CEF specific proteins and features. 

## FERREDOXIN DIVERSITY REGULATES ELECTRON PARTITIONING IN MAIZE

Maize has 4 genes encoding classical photosynthetic Fd proteins, FdI, FdII and FdV [57, 58] and a related FdIX sequence (Fig. **1a**). Of these, the mature sequences of FdV and FdII are highly similar (93% identity), while FdI is slightly different (88% and 89% identity to FdII and FdV respectively). FdV is not detectable at the protein level [57], while FdI is predominantly expressed in MC [59], and FdII is one of the most differentially abundant proteins between MC and BSC chloroplasts, being almost exclusively present in the BSC [59, 60]. This dramatic difference in distribution between chloroplasts performing mainly LEF and those conducting mainly CEF means that, *in vivo*, FdII must be almost exclusively transferring electrons in CEF. This could simply be an accident of expression, or it could also be because the FdII protein has specific properties that favor preferential electron donation to an acceptor involved in CEF, over support for NADP^+^ photoreduction. Maize *FdI* and *FdII* were heterologously expressed in a *petf* background of the cyanobacterium *Plectonema boryanum* [61]. In this case, the most abundant native photosynthetic Fd is absent, and the introduced proteins catalyze electron transfer in PET. *P. boryanum* cells expressing *FdII* had much higher ATP/ADP ratios, and much lower NADPH/NADP^+^ ratios than those expressing *FdI*, which resembled *P. boryanum* rescued with the native Fd (*PetF*) [61]. This output is identical to that expected in conditions of elevated CEF, which ensures low reduction of NADP^+^ and high generation of a pH gradient, driving rapid ATP synthesis. The authors hypothesized that decreased interaction of Fd with FNR leads FdII to donate electrons to CEF by default. The contribution of FdII to cyclic electron flow in maize is included in (Fig. **2b**).

The data from maize therefore indicate that it is possible to alter electron partitioning between CEF and LEF simply by changing the relative expression of specific *Fd *genes. Our understanding of the physiological roles of these different Fds was greatly helped by the extreme nature of PET in the maize BSC and MC, which are specialized for CEF and LEF respectively. C3 plants, algae and cyanobacteria also perform both LEF and CEF, and it is still not clear how they can regulate the flux through these pathways in response to environmental stimuli. In this review we will describe how findings from the work on the C4 system can help drive our understanding of this phenomenon in C3 chloroplasts. In particular we will address the question: Could alterations in the abundance of Fds specific for CEF and LEF also be part of a regulatory mechanism in C3 and algal chloroplasts and in cyanobacteria?

## ARE CEF SPECIFIC FD ISO-PROTEINS PRESENT IN OTHER PHOTOSYNTHETIC ORGANISMS?

The presence of genes for at least two different photosynthetic Fd iso-proteins in many C3 species [62, 63], algae and cyanobacteria shows this is hypothetically possible (Fig. **1A**). Data from cyanobacteria and algae is extremely limited, but there are several pieces of evidence from C3 plants that suggest this could be the case. Under normal light conditions, the flux through LEF would be expected to far exceed that through CEF [64, 65], and therefore Fds specialized for LEF should theoretically be much more abundant (major forms) than Fds specialized for CEF (minor forms). Indeed, major and minor photosynthetic Fds have been identified in several species, with Arabidopsis and pea the best studied examples. In both cases there is a more abundant, major iso-protein and an iso-protein that is only present at very low levels [8] or is undetectable [66-68]. Studies on gene expression and protein abundance have found that transfer of plants to conditions where CEF is necessary, such as high light or drought, causes increased expression of the genes for minor iso-proteins [69], and in the case of pea the Fd iso-form that is not detectable under normal growth conditions increases in abundance to become the dominant form [66]. These data therefore correlate activity of the minor photosynthetic Fd iso-protein with increased demand for CEF.

There are several genetic studies that also hint at a specific function for the minor photosynthetic Fd in CEF. Firstly, a comparison of chlorophyll fluorescence measurements between Arabidopsis lines with RNAi knock-down showed that decreased contents of either AtFd1 (minor iso-protein) or AtFd2 (major iso-protein) had opposite effects [63]. Knock-down of the major iso-protein had the anticipated effect of causing decreased electron transport rates and a build-up in excess reduction pressure in the PET chain, due to a lack of acceptors at PSI [63]. By contrast, knock-down of the minor Fd was not additive, and had the opposite effect. Electron transport rates actually increased slightly, and the abundance of acceptors at PSII indicated that the PET chain was actually in a more reduced state [63]. Such a phenotype, with electrons being lost from the PET chain, would be consistent with a decrease in CEF.

In addition, there are two studies on the transplastomic introduction of Fd into tobacco [62, 70]. The genes concerned encode a major Fd iso-protein from Arabidopsis (*AtFd2*) and a minor Fd from pea (*PsFd2*). Over-expression of the major and minor forms has dramatically different effects. The phenotype on over-expression of a major Fd is mild, with altered PET only observed following growth in the absence of CO_2_ [70]. By contrast, over-expression of a minor Fd causes a dramatically stunted phenotype, with several PET features typical of increased CEF: rapid induction of NPQ and slow induction of PSI oxidation on transfer to the light, a more oxidized state of the PSI active site, P700, at steady state, and rapid re-reduction of the P700 when actinic light is switched off [62]. It was concluded that the severe, stunted appearance of tobacco over-expressing the minor Fd was due to enhanced CEF, causing build-up of an excessive proton gradient to pH values that inhibited the Cytb_6_*f*. This would result in overall inhibition of PET and therefore photosynthesis [62]. Although an ideal study would involve a comparison of two Fds from the same species, this comparison between introduction of minor and major Fds to tobacco does provide strong evidence for the specific involvement of minor Fd types in CEF [62, 70]. 

There remain, however, several problems with this hypothesis, the most obvious being that in many cases the two Fds in a genome have much higher similarity to each other than to any other Fd in the database [62, 63] (Fig. **1a**). It therefore remains impossible to use sequence comparisons in order to identify any conserved feature, which might confer a biochemical or structural property allowing a minor Fd iso-protein to promote CEF. It seems likely that if specific Fds are involved in CEF, they are the products of separate gene duplication events, followed by selection for greater interaction with either PGRL1/PGR5, the NDH-complex, or FNR bound to Cytb_6_*f*. We know from the maize BSC / MC system that this has happened at least once, and when this knowledge is applied to the minor /major photosynthetic Fds found in some C3 plants it seems very likely that this has been replicated multiple times. Unfortunately, the lack of data on differences in PET activity of cyanobacterial and algal Fds means we are not certain if this is limited to higher plants. 

## POSSIBLE REGULATION OF CEF BY FNR LOCATION

As the catalyst for electron transfer from Fd to NADPH the activity of FNR will clearly effect the partitioning of electrons between LEF and CEF, irrespective of whether it is directly involved in CEF [34, 35] or not [36]. 

In cyanobacteria, the endosymbiotic ancestors of chloroplasts, FNR is bound close to the PET chain by a linker to the phycobilisome [71]. FNR remains membrane bound under photosynthetic conditions [40] and FNR association with the phycobilisome is essential for CEF [72]. However, under non-photosynthetic conditions, when FNR is required to transfer electrons in the opposite direction, from heterotrophically generated NADPH to support Fd-dependent metabolism, the enzyme accumulates in the soluble phase [40]. This is enabled by initiation of transcription at an earlier start site [73] resulting in a transcript with an alternative translation start site [42] – producing a shorter protein, which lacks the phycobilisome linker and is therefore soluble [42]. In addition, no association of a cyanobacterial FNR with the Cytb_6_*f* complex was been detected [39]. These data suggest that FNR plays no significant role in the transition between LEF and CEF in cyanobacteria, although it is possible that in conditions where high CEF is required, release of FNR from the thylakoid would allow greater interaction of Fd with the NDH complex [33]. Little is known about FNR:membrane association in algae, and algal FNR enzymes appear to be more similar to higher plant root type than leaf type enzymes (Fig. **1B**). This suggests that membrane attachment might be through a different mechanism than in higher plant chloroplasts. FNR has been isolated with Cytb_6_*f* and PGRL1 as part of a supercomplex involved in CET [28], indicating that dynamic recruitment of FNR to different locations in the chloroplast likely forms part of a mechanism for regulating PET. In higher plants the location of FNR – one of the most basic and earliest identified components of PET [7], remains controversial. FNR seems to be a relatively “sticky” enzyme, and has been reported as a subunit of several major PET complexes including Cytb_6_*f* [39, 41], PSI [74], and the NDH complex [38, 75]. 

Great advances have been made in the last 5 years, with the identification of two, dedicated FNR binding proteins at the thylakoid membrane of higher plant chloroplasts: the Tic62 protein, which is also a component of the inner envelope translocon [76], and the TROL (thylakoid rhodanase like) protein [77]. Although Arabidopsis mutants lacking Tic62 show no strong phenotype [78], knock-out of TROL does result in perturbed PET [77]. It has been suggested that these binding proteins may simply retain FNR at the membrane to stabilize it against degradation, and that its release, as a soluble enzyme is associated with its activity in PET [79]. It has also been shown that a large proportion of FNR in the chloroplast is not strongly bound to the membrane, and is either soluble, or very weakly associated [41, 44]. Membrane association of FNR is also regulated in higher plant chloroplasts, and the association of FNR with the Tic62 protein has been shown to be pH dependent, with weaker binding at the higher pH values associated with active PET [80]. In addition, the structure of the FNR N-terminal has been shown to be important for FNR recruitment to the membrane [81], and is even able to determine the complex to which FNR is recruited [81]. Moreover, regulated truncation of the N-terminal has been shown to up-regulate FNR binding to the membrane [82].

The regulated release and recruitment of FNR to higher plant thylakoid membranes and its ability to interact with different membrane complexes has led to suggestions that redistribution of FNR could be integral to regulating changes in electron partitioning in higher plants, as well as in algae [35]. Indeed there is evidence to suggest that plants with decreased FNR contents have stimulated electron partitioning into other Fd-dependent pathways, such as nitrite assimilation [83], and that they are impaired in photosynthesis in general [84] and CEF in particular [35]. Most higher plants are found to have at least two photosynthetic FNR iso-proteins [41, 44-46] (Fig. **1B**), but unlike Fd, these are clearly divided into two conserved clades – one with a more basic, and one with a more acidic isoelectric point [44, 85]. These different proteins show no difference in their electron transfer ability between NADPH and Fd [41, 44], but they do show variation in their ability to bind to the membrane [41, 44]. 

## CAN THE FINDINGS ON BUNDLE SHEATH FNR BE APPLIED TO OTHER CEF SYSTEMS?

In order to determine whether the location of FNR can influence partitioning into LEF and CEF in C3 plants, it could be helpful to examine FNR properties in the different maize cell types specific for LEF and CEF. This information is summarized in Fig. (**1**). In maize there are 3 FNR iso-proteins, and interestingly, these are unevenly distributed between the cells conducting CEF (BSC) and those performing mainly LEF (MC). FNR1 is bound exclusively to the membrane [41] and seems to be evenly distributed between the BSC and MC [81, 86]. FNR3 is only found as a soluble protein [41] and is mainly detected in the MC [81]. FNR2 is present in both membrane and soluble fractions, and is much more abundant in the MC than the BSC [60, 81, 86]. The result of this distribution of FNR between the cell types is that the MC contains some membrane bound FNR, but the majority of FNR is recovered in the soluble fraction [81]. By contrast, the BSC lacks soluble FNR, with most of the protein strongly membrane associated [81]. Both FNR1 and FNR2 were found associated with the Cytb_6_*f* in maize [41], and bind specifically to TROL and Tic62 when expressed in Arabidopsis [81]. However, the maize Tic62 protein does not contain an FNR binding domain, and so it must be assumed that binding to TROL and Cytb_6_*f* is adequate for both types of PET in maize. Considering the differentiation of MC and BSC in PET, it therefore seems possible that tight FNR binding to the membrane favors CEF, while weak binding / soluble FNR is necessary for LEF, with the capacity for dynamic regulation.

It is still unclear exactly how FNR influences electron partitioning around the PET chain in C3 plants, and which FNR associations contribute to LEF and CEF. It has been suggested that the real catalyst of NADP^+^ photoreduction might be soluble FNR [79], which would be consistent with the abundant soluble FNR found in the maize MC [81], but previous studies have reported that NADP^+^ photoreduction is more efficient when the enzyme is bound to the membrane [43] and that FNR free thylakoids cannot replicate wt rates of NADP^+^ photoreduction, even when very high concentrations of soluble FNR are added [83]. In transgenic Arabidopsis (C3) plants with enrichment of FNR at TROL, there was a more rapid induction of NPQ on transfer to the light, and a higher ratio of PSI/PSII excitation, which would be consistent with enhanced CEF. In combination with the lack of soluble FNR in maize BSC, this could be an indication that membrane bound (and specifically TROL bound) FNR is specifically involved in CEF. However, the phenotype of TROL knock-out mutants seems to be mainly affected in LEF rather than CEF [77]. The contrasting findings from different species [77, 78, 81, 82] indicate that there may be species specific differences in the regulation of FNR – membrane association.

## CONCLUSION

In conclusion, data comparing PET in the MC and BSC cells of maize, which are specialized for LEF and CEF respectively, allow us to speculate about how the transition between these two different PET pathways could be regulated in other photosynthetic organisms. The actions of Fd and FNR at the end of the PET chain are pivotal in determining the fate of photosynthetically generated electrons: either their release into soluble metabolism, or return to the PQ pool, in order to increase the ratio of ATP/NADPH production. It is now clear that in MC and BSC, different Fd iso-proteins help to control the partitioning of electrons into these two pathways, and these findings have informed investigations to identify equivalent proteins in C3 plants. The duplication of photosynthetic Fd genes in other photosynthetic organisms, including algae and cyanobacteria, indicates that duplication of a Fd gene, followed by subsequent mutation to favor electron partitioning into CEF, might be a mechanism for regulating PET that has been frequently repeated through evolution. In addition, the strong membrane association of FNR in the BSC seems to correlate with increased CEF. It is to be hoped that this finding will help inform studies on C3 plants, into the potential role of membrane bound FNR in CEF. Although the mechanisms of FNR: membrane recruitment are very different in algae and cyanobacteria, there is evidence that recruitment to different supercomplexes is involved in regulating LEF/CEF partitioning in *Chlamydomonas* [28]. This is probably not the case in cyanobacteria, where the membrane: soluble transition is more associated with the transition between photosynthetic and heterotrophic metabolism. 

## LIST OF ABBREVIATIONS

BSC: = Bundle sheath cell
CEF: = Cyclic electron flow
Cytb_6_*f*: = Cytochrome b_6_*f* complex
Fd: = Ferredoxin
FNR: = Ferredoxin:NADP(H) oxidoreductase
LEF: = Linear electron flow
MC: = Mesophyll cell
NDH: = NAD(P)H dehydrogenase complex
NPQ: = Non-photochemical quenching
O_2_^∙-^: = Superoxide
PC: = Plastocyanin
PET: = Photosynthetic electron transport
PGR5: = Proton gradient regulation 5
PGRL1: = PGR5 like 1
PQ: = Plastoquinone
PSI: = Photosystem I
PSII: = Photosystem II
